# The regional training centre for the emergency medical teams initiative in the WHO African region: a review of the development and progress over the past 4 years

**DOI:** 10.3389/fpubh.2025.1542261

**Published:** 2025-03-28

**Authors:** Boniface Oyugi, Lazaro Gilberto Martinez-Monterrey, Leilina Ayalew, Joseph Chukwudi Okeibunor, Jerry-Jonas Mbasha, Rashidatu Kamara, Pryanka Relan, Nahom Tadelle, Alegnta Gebreyesus, Neima Zeynu, Flavio Salio, Thierno Balde, Fiona Braka, Abdou Salam Gueye

**Affiliations:** ^1^World Health Organisation, Emergency Medical Teams Initiative, Geneva, Switzerland; ^2^Centre for Health Services and Clinical Research, University of Hertfordshire, Hertfordshire, United Kingdom; ^3^World Health Organisation, Country Office, Addis-Ababa, Ethiopia; ^4^World Health Organisation, Emergency Preparedness and Response Programme, Regional Office for Africa, Brazzaville, Republic of Congo; ^5^World Health Organisation, Emergency Preparedness and Response Programme, Regional Hub for West Africa, Dakar, Senegal; ^6^Ethiopian Emergency Medical Team, Ethiopian Public Health Institute (PHEM), Addis Ababa, Ethiopia

**Keywords:** emergency medical teams, training, training centre, WHO African region, public health emergencies, policy cycle

## Abstract

**Background:**

A coherent and systematic approach to education and training of the workforce under the EMT initiative has been identified as an imperative step to improve the quality and professionalism of emergency response teams. On April 14 2021, the WHO and the Ethiopian Ministry of Health (MoH) jointly launched a training centre to enhance the delivery of emergency medical and health services in the continent when faced with humanitarian and other public health emergencies (PHEs).

**Objective:**

This paper describes the development and progress of the EMT training centre in the WHO African Region over the past 4 years and elucidates its implementation processes. It further elucidates the lessons learned, including the complexities and challenges, and proposes recommendations for enhancing the centre’s future.

**Methods:**

This descriptive retrospective study systematically documents the development and progress of the EMT training centre in the WHO African Region over the past 4 years. The study applied a mixed-methods qualitative approach through key informant interviews (KIIs) and document reviews. The study utilises the policy cycle framework as an analytical framework focusing on the EMT agenda setting (problem identification), formulation, adoption, implementation, and evaluation.

**Results:**

The concept emerged at the outset of the pandemic, driven by the need to establish a United Nations (UN) field hospital for evacuating UN staff. Addis Ababa was chosen for its strategic location, accessibility, and strong political support. However, the idea evolved into a training centre based on the decision not to include Addis Ababa in the UN staff safe and rapid patient transfer and medical evacuation (medevac) system. Following the scoping mission, the centre’s design and the training portfolio were done, and implementation started immediately following the joint official launch by the WHO and Ethiopian MoH. Since implementing the training centre concept, 12 countries in 2022 and 7 in 2023 benefited from different training out of the 10 countries prioritised at the onset. Continuous refinement of the procurement has happened throughout the implementation process. In October 2021, a comprehensive monitoring and evaluation framework with indicators and tracking timelines was developed at the inception of the training centre.

**Conclusion:**

The future of the centre will not only be limited to EMT but will also serve as a training centre capable of hosting various types of training and technical topics that could be useful, such as training on Rapid Response Teams (RRTs) and the WHO Regional Office for Africa (AFRO) AVoHC-SURGE initiative, simulations, etc. It is envisioned as a knowledge hub, a place where the region and the countries could work together to improve effectiveness of their activities and interoperability, develop communities of practice, generate research ideas, or share knowledge on documentation processes and existing resources.

## Introduction

Over 100 health emergencies are experienced yearly in the African Region (1), making it the most heavily impacted region worldwide. Currently, the World Health Organisation (WHO) Regional Office for Africa (AFRO) faces the daunting task of managing over 142 ongoing public health emergencies (PHE), including disease outbreaks and humanitarian crises, solidifying its position as the global epicentre for public health challenges (1). The Region’s PHE often proves overwhelming for weakened health systems, interrupting essential health services and disrupting societies and economies (2, 3). However, many of these emergencies are preventable or controllable through proven public health interventions; some include working with the communities, emergency medical teams (EMTs), and simple, available, and innovative strategies such as phone and internet technology.

The COVID-19 pandemic uncovered the essential need for Africa’s self-reliance. A WHO analysis revealed that less than 10% of countries in the African Region possess the necessary workforce to effectively prepare for, detect, and respond to public health risks (4). The workforce challenge highlighted by the pandemic underscores the urgent need for the continent to cultivate national elite responders, build robust surveillance systems, and invest in pandemic preparedness. These measures would strengthen response capabilities and contribute to a more secure future. Consequently, it is imperative to prioritise enhanced planning and preparedness for emergency management and mobilise external healthcare professionals for direct clinical care and capacity-building support. This presents an opportunity to improve preparedness, particularly in the areas of training, emergency workforce development, and community collaboration.

The EMT initiative works to fortify the national surge capacities and facilitate the deployment of internationally classified teams of healthcare professionals to countries and territories during emergencies, especially in outbreaks and natural disasters, to provide immediate assistance when national health systems are overwhelmed (4). It was endorsed as crucial for maximising emergency response to large-scale health emergencies at the World Health Assembly in 2015 under the Global Health Emergency Workforce (GHEW) (5). The initiative was officially introduced in the WHO AFRO Region in 2017, making it the last region to implement it (6). However, efforts are underway to develop and strengthen EMTs within the region.

A coherent approach to the education and training of the workforce under the EMT initiative has been identified as an imperative step to improve the quality and professionalism of emergency response teams. Several organisations and training institutions have instituted education and training programmes for PHE responses, but there is a need for more standardisation of scope, curriculum and quality. The lack of common education, training design, and provision standards has been highlighted. Some proposed training models focus on individuals rather than multidisciplinary teams. The WHO EMT secretariat is mandated to manage training, capacity building, standard setting and quality assurance processes for the global EMT Initiative and to support countries and regions in coordinating response operations (7).

On April 142,021, the WHO and the Ethiopian Ministry of Health (MoH) jointly launched a training centre envisioned to bolster the delivery of emergency medical and health services in the continent when faced with humanitarian and other public health emergencies (PHEs). The centre was established on the premises of the Addis Ababa Field Hospital to address the need for everyday preparedness and build a multilayer surge capacity by establishing self-sufficient national EMTs that adhere to international standards and building in-country response capacities.

This paper describes the development and progress of the EMT training centre in the WHO African Region over the past 4 years. It further elucidates the lessons learnt, including the complexity and challenges, and proposes recommendations for enhancing the centre’s role and its future.

## Methods

### Analytical framework

This systematic analysis utilises the policy cycle model to show how the EMT training centre in the WHO African Region evolved and how its implementation developed or happened over the past 4 years. The goal is to draw lessons from each process. The policy cycle was chosen for examining the subject in this paper because there are distinct lessons or perspectives among different actors about how the formulation and implementation of the model followed a standard sequence of steps reminiscent of the policy cycle. The policy cycle is integrated into the analysis as it offers a structured process depicting how issues or problems are framed and navigated through a series of actions, ultimately guiding the resolution of the problem (8). The term ‘Policy cycle’ refers to recurrent cycles and procedures that eventually culminate in the development of public policies (9). First proposed by Harold Lasswell (10) and subsequently adopted by others [e.g., Brewer (11); Jenkins (12); deLeon (13); Bridgman and Davis (14); Mohammed (8)], the policy cycle has undergone several iterations and modifications resulting in the division of the process into a standardised sequence of key stages: agenda setting (problem identification), policy formulation, adoption, implementation, and evaluation. This study adopts these five standard stages to analyse the EMT training centre (Table 1), and the subsequent paragraphs will summarise the cycle’s steps, addressing the discussion’s specific aspects.

**Table 1:** Analytical framework.

Stages	Meaning	Potential questions
Agenda setting (problem identification)	Identifying a problem that requires interventions. It arises whenever individuals or groups (mass media, interest groups, citizen initiatives, and public opinion) call for action on a situation (8, 41). It lays the foundation for all successive stages by ensuring policymakers understand the problem, its scope, and its impact on stakeholders. This understanding informs the agenda setting, where priorities are determined, and specific problems are selected for policy development.	What brought out the need for the EMT training centre?
Policy formulation	It is a crucial process of exploring available options and alternative proposals for action. It involves defining, accepting, or rejecting options on a subject matter of formulation (8, 14).	How was the concept of the EMT training centre formed? What were the alternatives, and were they evaluated? How? What were the envisaged contents?
Policy adoption	It is the decision-making stage where policy actors issue statements of intent to undertake. It includes deliberations and debates leading to selecting and enacting the preferred choice (8, 10).	At what point was the EMT training centre adopted? How was it taken up or launched?
Policy implementation/ intervention	It is carrying out the programme activities to achieve its goals. Some researchers refer to it as translating plans into actions or putting solutions into effect (10).	What are some of the lessons from the implementation of the policy? Who were the actors, context and the processes involved?
Policy evaluation	It is the process of appraising, rating, and assessing the outcomes of the policies (42).	What was the implementation or the progress of the EMT training centre evaluated? How and what were the outcomes?

### Study design

This study employs a descriptive retrospective design with a mixed-methods qualitative approach to systematically document the development and progress of the EMT Training Centre in the WHO African Region over the past 4 years. By integrating qualitative data sources, the study captures a comprehensive narrative of the centre’s establishment, evolution, and contributions, highlighting key milestones, contextual influences, and operational challenges. The retrospective nature of the study allows for an in-depth exploration of historical trends and policy decisions that shaped the centre’s trajectory, while the qualitative focus ensures nuanced insights into stakeholder perspectives and the impact of training initiatives on EMT’s capacities across the region.

### Data collection and analysis

The study employed a mixed-methods qualitative approach, combining key informant interviews (KIIs) and document reviews. The KIIs were conducted with individuals directly involved in the formulation and implementation of the EMT Training Centre in the WHO African Region. These participants, referred to as “insiders,” provided valuable insights into the processes, dynamics, and strategies underpinning the centre’s development. A total of four KIIs were conducted between October 2022 and January 2023, using semi-structured interview guidelines. The interviews focused on key aspects such as the conceptualisation of the training centre, its implementation journey, and future prospects, offering a rich understanding of its evolution and impact.

For the document review, we included materials written in English, French, and Portuguese, encompassing published studies, grey literature, reports, and media coverage related to the EMT Training Centre. The document sources consisted of: (a) secondary data provided by WHO teams in the African Region and headquarters, such as mission reports, assessments, surveys, monthly bulletins, and meeting minutes pertaining to the EMT Training Centre’s implementation; (b) key resources from WHO EMT websites; (c) media coverage from local and international outlets; (d) direct correspondence with the Ethiopia EMTs for data on their contributions; and (e) comprehensive internet searches. PubMed searches used the keywords “emergency medical team OR Emergency medical teams OR EMT” combined with “Africa OR sub-Saharan Africa OR [all the 47 WHO African countries]” and “training centre.” Articles were included if they met the criteria of being in the relevant languages, addressing EMT implementation, and published between 2017 and June 2024. The PubMed search yielded 270 studies, which were screened for eligibility. Exclusions were made for articles not relevant to the EMT Training Centre. After reviewing titles and abstracts, 11 studies met the criteria. A detailed full-text analysis further narrowed this to three articles (6, 15, 16) that were subsequently included in the analysis.

The content of the review was analysed using a framework analysis approach, as outlined by Gale et al. (17). This approach was guided by an *a priori* analytical framework developed to systematically organise and interpret the data. Specifically, the analysis process began with familiarisation, where all data, including transcripts and documents, were reviewed in detail to gain a comprehensive understanding of the content. Following this, initial coding was performed by identifying recurring themes, patterns, and key concepts within the data. These codes were then mapped onto the pre-defined categories of the analytical framework, which included thematic areas such as public health capacity building, emergency response training, and alignment with regional public health strategies.

This study represents systematic documentation of a programme conducted by the authors as part of routine monitoring and evaluation of EMT activities within the WHO African Region. Formal ethical clearance was not required, as the data were anonymised or aggregated, with no collection of sensitive personal information. All focus group participants provided informed consent, and principles of confidentiality and voluntary participation were strictly observed throughout the study.

To ensure the robustness of the findings, triangulation was employed by cross-referencing data from multiple sources, including interviews and document reviews, thereby validating insights across perspectives. The analysis process was iterative, allowing categories and themes to be refined as new insights emerged. The final analytical framework was collaboratively reviewed by the research team to ensure consistency and reliability, with discrepancies resolved through discussion. This systematic and rigorous approach underscores the validity of the findings and their relevance to the study’s objectives.

## Results

### Agenda setting (problem identification)

***There was an ongoing need to develop a concept to mitigate some of the challenges faced by the healthcare systems on the continent despite having extensive experience in responding to emergencies and the need to set up evacuation centres globally.*** The healthcare capacity in the WHO African Region has long been recognised as vulnerable, underfunded, understaffed, and underequipped, posing significant challenges to emergency preparedness and response. A systematic analysis of healthcare infrastructure across the region revealed severe shortages, with an average of 0.97 ventilators and 3.10 ICU beds per 100,000 people, alongside just 2.42 anaesthesia providers per 100,000 people (18, 19). At the onset of the COVID-19 pandemic, the situation was even more alarming—43 countries in the region collectively had fewer than 5,000 ICU beds, translating to only five ICU beds per 1 million people (19, 20). Additionally, public health services in 41 reporting countries had fewer than 20,002 functional ventilators, leaving a vast population underserved during critical health emergencies. Workforce shortages compounded these challenges; in 2018, the region had only three medical doctors per 10,000 persons and 10 nurses or midwives per 10,000 persons (21). These stark gaps underscored the urgent need for strengthening emergency medical response systems, particularly through EMTs. The onset of the COVID-19 pandemic acted as a catalyst, accelerating the adoption of EMTs as a strategic intervention to enhance emergency preparedness and response across the region. This reinforced the importance of structured, well-equipped, and well-trained teams to bridge critical gaps in healthcare infrastructure and workforce capacity.

At the onset of the pandemic, the United Nations (UN) system engaged in internal discussions and debates on how to effectively manage staff in country and regional offices who might contract COVID-19 and develop severe illness. A key focus of these discussions was the logistics of evacuating UN staff and patients from temporary structures worldwide, particularly in regions where health systems were already under significant strain. Several teams at WHO Headquarters (HQ), including the EMT Secretariat, the Case Management Team, and the Staff Health and Well-being Unit, played a pivotal role in shaping policies to ensure the safety, medical support, and well-being of WHO personnel globally. These discussions helped develop a structured approach to staff health management, ensuring that contingency plans were in place for emergency medical evacuations and access to critical care during the crisis.

***A working group was set up to examine staff health and medical evacuation.*** Discussions were held about setting up temporary structures worldwide in various strategic areas. The structures were envisaged as UN field hospitals for all United Nations staff who needed a higher level of care than was available in the country where they were working.

***At the onset of the discussions, the government of Ethiopia was already starting to develop and build a similar national structure called the Millennium Centre, which was a COVID-19 centre for the population.*** So, political buy-in came in handy, there were thoughts and discussions about utilising the same resources, sharing ideas, and utilising the same for the UN structure. The country was willing to share resources and ideas and support the hosting of this centre. Importantly, at the onset, there was also political buying internally at the UN, including the World Food Programme (WFP) and all of the agencies, UNICEF, and many UN agencies that were engaged because this was supposed to be a hub for receiving UN staff.

### Policy formulation

***There was a proposition of a plausible location for the centre.*** In February and March 2020, discussions within the UN proposed that the WFP, known for its strong operations, logistics and equipment management, would design a field facility and then offer it to evacuate UN staff in the African Region in Addis Ababa. Addis Ababa was chosen because it was geographically strategic and considered one of the UN COVID-19 safe and rapid patient transfer and medical evacuation (medevac) location options according to the Global Humanitarian Response Plan (GHRP) reference. At the time, there were many COVID restrictions, and Addis Ababa was one of those hubs that were easy to access and depart from regarding COVID-19 and visa restrictions. These were the main factors considered from an evacuation standpoint. Other options were also considered:

*“…so there was there was consideration about where to set it up…there was consideration around the different regions…other considerations from other countries…there were discussions to have other ones in West Africa, South Africa, but we just decided to start with Addis, and…there were discussions at the country level that was they were willing to host it.” Respondent 01.*

In early April, the Federal Government of Ethiopia, through the State Minister of Finance, graciously indicated its acceptance of WFP’s proposal to construct a dedicated COVID-19 field hospital to provide care for United Nations and international personnel from the NGO community. At the end of April, the Addis Ababa City Administration officially handed over 25,000 m^2^ of land to the MoH to establish the field hospital with a planned capacity of 92 beds.

***Discussing the contractual process and delays in interagency agreements.*** Within the UN, there were extensive debates about the contractual procedures and the allocation of responsibilities, particularly regarding the hospital’s operation and the modus operandi. The UN believed it should take the lead since it targeted UN staff, while the WHO would provide technical assistance. This discussion phase required significant time for the various teams to reach a consensus, and it was fraught with political undertones. WFP set up the structure.

***Internal discussion to develop a training centre based on the field hospital.*** Subsequently, in April and May 2020, after deciding not to include Addis Ababa in the UN staff medevac system, the option of utilising the already constructed facility as a regional Emergency Medical Team Training Centre (EMT-TC) was presented. The EMT secretariat at the WHO headquarters received internal approval to collaborate with other UN agencies and transform the field hospital into a training centre, thereby saving time. Therefore, a meeting was arranged among the WHO HQ, the AFRO, and the WHO Country Office (WCO) of Ethiopia, as they had identified the need for such a centre. Although there had been preliminary discussions regarding establishing training centres, no concrete steps have been taken thus far. The discussions were still early, but the opportunity to utilise an existing field hospital without Human Resources (HR) seemed like a perfect fit for combining the two concepts: a training centre and a field hospital. This combination offered a secure environment for training that included various simulation techniques and on-the-job training, in addition to addressing the local community’s need for additional bed capacity (22).

***Developing the design and the training portfolio, including the training manual.*** The initial training discussions were centred around who to train and what training to organise. The aim was to broaden the training beyond EMT-related topics and to address various clinical and technical areas, with a particular focus on the ongoing challenges faced by the region, such as Ebola (23). During the meeting, it was agreed that establishing training centres was necessary to enhance the technical and operational skills and knowledge of EMT members and other clinical care management personnel using simulation-based and team approaches. These would not replace the training at the country level but have a way in which countries would be facilitated to share experiences:

*“The idea was not to replace the needed trainings at the country level but rather complement them adopting a ToT [Training of Trainers] approach in the centre. Having more countries together that will be facilitated to share their experiences. Incorporate a simulation component that can bring some competition within the workforce to augment the training, thereby building up the team on more targeted technical skills.” Respondent 04.*

In the long term, the goal was to augment the timeliness and quality of health services provided by national and international EMTs during all health emergencies. This plan also aimed to strengthen the capacity of national health systems to lead and coordinate emergency responses. The case management team initially proposed training healthcare workers from the region on ongoing clinical guidelines, specifically for COVID-19 and other emergencies. Steps would include analysing the needs and then targeting the prioritised countries. After that, they will design their road map incorporating the training component. Eventually, it was decided that a scoping mission was necessary to assess the feasibility of the envisioned training centre.

The proposition was to deliver potential training products onsite in three main categories: short-focused products, one-day options for thematic-specific areas, and multi-day options as flagship events. The expected training portfolio would be wide and organised per pathways and thematic areas such as clinical, water sanitation and hygiene (WASH), logistics, general, surveillance, and others (23).^1^ The training portfolio was fine-tuned following the discussion with the Ethiopian MoH and subsequent meetings (24). The modules are shown in Table 2. The envisaged training focused on building trainer capacity and communicating the training pathway. The cascading Training of Trainers (ToT) approach was proposed to promote the participatory adult learning process of having a core group of trainers to manage the implementation of training and leverage the regional/ global EMT network and local expertise. In addition to classroom-based presentations and discussions, major emphasis was placed on practical field exercises. The second element was prioritising the training based on regional needs. For instance, burns and mass casualty management were highlighted as gaps in the regions. It would also include adding the research component and looking into possible testing of innovations at the centre with a couple of ideas looking at their investment worthiness.

**Table 2:** Proposed training modules.

Module	Purpose of the training	Hours	No. of participants
General training module			
EMT awareness session	To sensitise national health authorities and other stakeholders about the advantages of having national EMTs in their countries.	24	30
In-depth training modules			
EMT team member (TM) induction	To provide a general overview of the scope of the EMT Initiative for National EMTs. To enlarge the roster of the country’s National EMT. To be the basis of the Implementation Workshop-Training to be held immediately after the Induction Training. To offer the basis for each EMT pillar, which will be developed later in specialised training.	30	30
10-step implementation	To bring together relevant stakeholders and decision-makers from the country’s health sector to create awareness of the needs and vision for implementing the National EMT. To develop a joint roadmap for implementing National EMTs, adapted to the country’s context.	24	30
EMT training of trainers (ToT)	The training aims to train a National Faculty team, who can then replicate training at the national level and support training across the region.	24	16
Pre-deployment training	The last generic training is done once the EMT is at an advanced stage of its formation and process. It is aimed at preparing the EMT for deployment.	24	30
SOP workshop	The training is done once the implementation process is triggered and aimed at discussing the experience of the team called to twin with the National EMTs.	38	30
EMTCC specialised modules			
EMT TM induction focused on the humanitarian crisis	An EMT induction training is stated above, specifically in a humanitarian context.	38	30
EMT TM induction focused on cholera case management (CM)	An EMT induction training, as stated above, specifically focused on cholera CM.	38	30
Case management and operation in burns mass influx	The regional ToT on the massive influx of mass casualty burn patients is strengthened using the capacitated national network of EMT.	38	30
Burn management specialised training	The regional ToT on the massive influx of mass casualty burn patients and specialised treatment is strengthened using the capacitated national network of EMT.	38	30
WASH/operation support and logistics (OSL)	It is specialised training that targets OSL/WASH focal points. It teaches specific and practical knowledge of OSL and WASH, allowing participants to move forward in implementation and self-sufficiency.	38	24
Resource mobilisation workshop	This training provides the main tools and skills to mobilise resources that will allow the team to be self-sufficient.	24	18

Source: Original concept note and steps in the implementation of EMT (23, 29).

The training was envisaged to be extended to 10 pilot countries, identified in consultation with the country’s MoHs, the WHO HQ, and AFRO. Additionally, it was initially planned that the training centre would be customised to meet the needs of each team based on a needs assessment and in accordance with the mentorship programme. This approach would enhance the technical skills of healthcare workers through research and innovation and adjust the training curricula to the specific requirements of each country. Moreover, countries would have access to training and simulation opportunities. They would receive support from WHO and classified EMTs to develop their capacities, including providing equipment through a twinning approach or other EMTs during the response phase through just-in-time training and equipment. The organisations that took part in this phase were the WHO (HQ and AFRO). The processes leading to this phase commenced during the scoping phase and are still ongoing (6).

***A scoping mission was organised to consider alternatives and suggest the way forward.*** A joint team of 9 members, including WHO HQ, WHO AFRO, WCO in Ethiopia and the Ethiopian MoH, was set up to conduct a scoping mission, which was conducted between 14 to July 16 2020 (6). The objective of the scoping mission was to assess the feasibility of establishing an EMT-TC using the temporary facility (tent structure) set up by the WFP and UN system for the initial purpose of a field hospital to ensure timely access to care to UN personnel in the African Region (22). It was also used to assess the facility, interact with key stakeholders, and propose a way forward.

Three possible options were identified for using the facility in Addis Ababa and setting up an EMT Training Centre.

1) To augment bed capacity, the current facility would be used as a Treatment Centre. This would entail either handing over the facility to the Federal Government of Ethiopia or allowing the UN system to own it while ensuring its management and running through a partner organisation. WHO was considered a possible partner in providing technical advice on required adaptations/ modifications and equipment.
2) Configure the facility to support access to emergency care services or treatment centres and training. This was presented as a flexible and scalable solution that was adaptable to the needs and the current epidemiological trend. It was considered to entail handing over the facility to the Federal Government of Ethiopia and the support of WHO to ensure the functionality and running of the training centre. WHO was considered a facilitator for providing equipment for the treatment centre area and identifying available EMTs to support the final setup and initial training needs.
3) To identify a new location for the training centre offering the same environment and training space to expose trainees to a fair approximation of real-life practice in a field hospital setting. However, possible on-the-job training could be already provided by the EMT Network at the Eka Kotobe, St Paul or St Peters hospitals as referral facilities for critical cases.

The findings and recommendations from the scoping mission were presented using the four S (Space, System, Supplies and Staff) approach. Plausible scenarios were proposed and costed for all four S except for the staff component, which was not quantified or costed. It included the recommendation of elaborate flows (footprints) of the installation, considering patients, personnel (both clinical and operational support staff), supplies, cleaning routes, waste management (from collection to storage and treatment area), and medical instruments/equipment separately. In addition, it considered operational support (power, fuel usage, generators and capacity, telecommunication, safety and security, water supply system, sanitation for staff and patients, and waste management) as well as equipment and supplies.

The challenges in treating COVID-19 critical cases were reported, suggesting a more prudent approach to setting up the treatment centre targeting critical cases and training through the EMT Network. The estimation included two important elements of uncertainty: the intended scope of the facility and the ratio of severe/ critical/ moderate patients for the treatment centre, as well as the consumption of each electrical item.

***Commitment and determination to adopt the policy and make it work as a training centre.*** Following WHO’s commitment through its Executive Director of the Emergency Programme to the Excellency Minister of Health and the interaction with relevant stakeholders during the scoping mission, there was a consensus on the significant relevance and opportunity the training centre represents at the national and regional levels.

*“After a scoping mission, which included a training person and a coordinator nominated by the WHO African Regional Office to carry it forward and make it quite strong and useful for the region in terms of training.” Respondent 01.*

### Policy adoption

**Figure 1:**
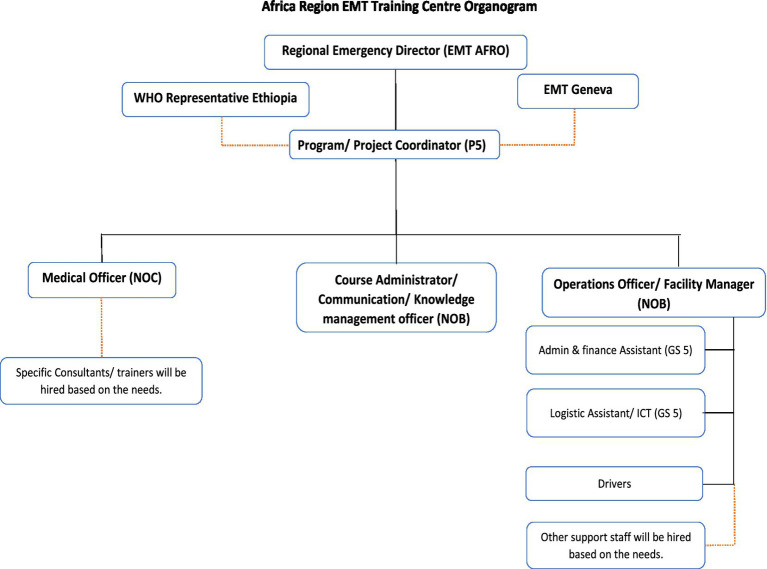
Proposed organogram from the onset.

***Organising the human resources to guide the next steps and the organogram.*** After the scoping mission, an organogram was developed to kickstart the project. The organogram contained a team that could run a massive training portfolio. It included a programme coordinator, somebody to manage the data, somebody to manage the logistics, somebody to run a training centre, and an administrative assistant. A project coordinator was proposed, identified and hired, whose role included guiding the next steps of the process based on the next steps in collaboration with the WCO to guide the next steps. The role included ensuring constant technical guidance and project oversight, facilitating alignment with other initiatives (e.g., WHO Academy), and discussing and involving key national and regional stakeholders (Figure 1).

***There was a lot of political and high-level buy-in regarding its running and commencing work.*** The Regional Director of the WHO AFRO and the top-level Ethiopian MoH were involved in the initiative. At the country level, Ethiopia was willing to take on the responsibility of hosting the training centre. On April 14, 2021, the WHO and the Ethiopian MoH officially launched the training centre to enhance emergency medical and health services delivery in the African Region during humanitarian crises (15, 25, 26). The centre was established at the Addis Ababa Field Hospital to address the need for ongoing preparedness and create a multilayer surge capacity by forming self-sufficient national teams that comply with international standards. The objective was to build in-country capacity and contribute to the EMT network.

**Figure 2:**
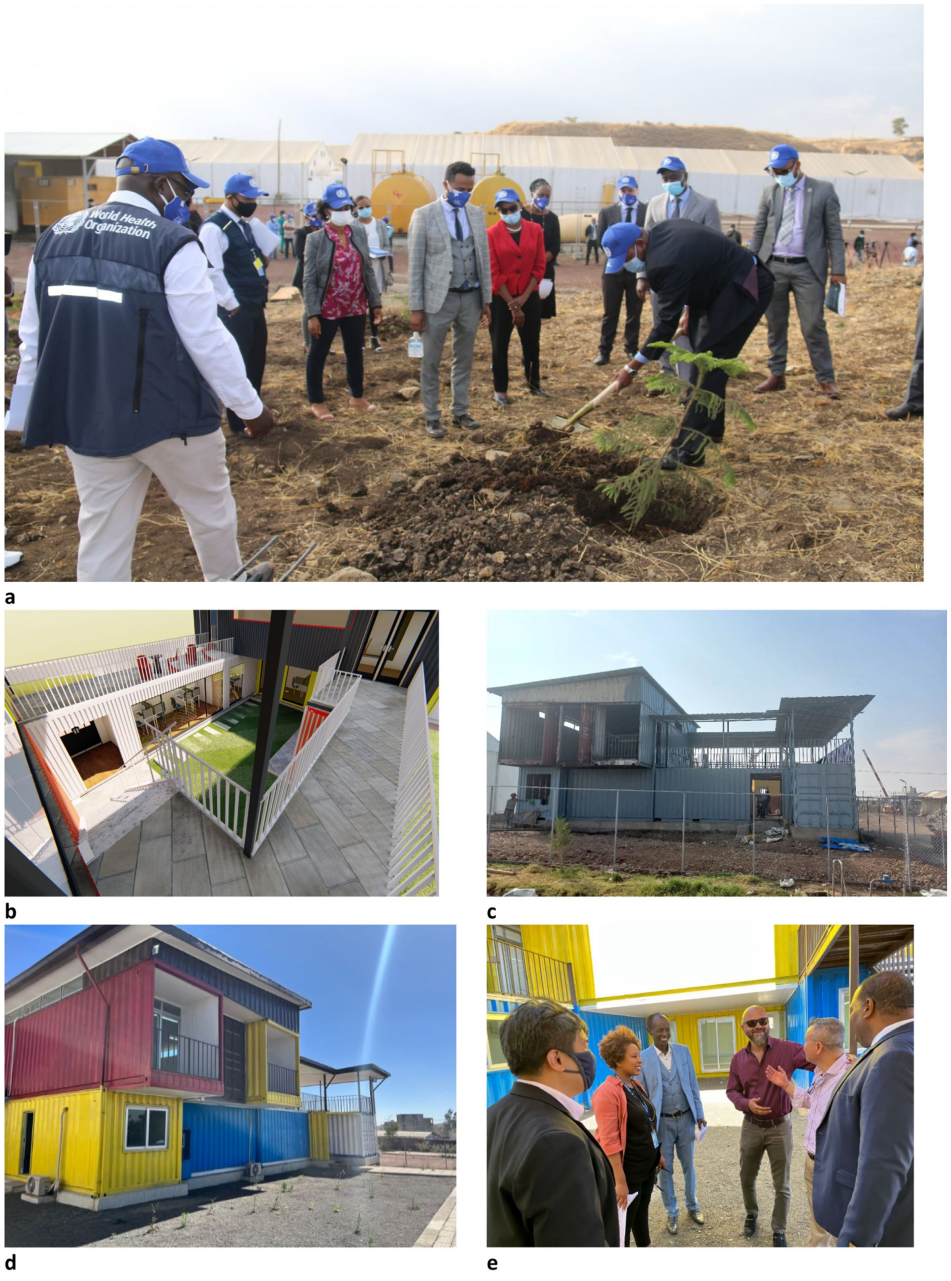
Representative photos showing the steps involved in the making of the EMT training centre. **(a)** Shows the joint launch of the training centre on April 14, 2021, led by WHO and the Government of Ethiopia; **(b)** The paper design of the training centre; **(c)** Set up and first phases of the construction; **(d)** Final set up and finished construction, **(e)** Launch and presentation of the centre. Source (25).

Subsequently, a memorandum of understanding (MoU) was drafted to solidify the collaboration between the government of Ethiopia and the WHO in operating the regional EMT-TC in the country (27). The roles and responsibilities of each party were outlined and summarised in Table 3, reflecting the progress made in implementing the initiative. The process of agreeing and signing is ongoing (Figure 2).

**Table 3:** Roles and responsibilities of the different parties in the MoU.

Roles of the MoH	Roles of WHO
Contribute financially on a co-share basis to support services and maintain common areas. Ensure the continuation of the current support services, including power and water, and timely communication related to possible outages. Ensure and facilitate the in-country arrival of members of the EMT Network and other collaborating partners in line with the scope of the Regional EMT training centre activities. Four (4) weeks in advance, inform WHO about the potential use of the facility for SURGE capacity purposes. Ensure land use of the EMT training centre; in cases of a change of location, the Ethiopian government will provide a substitute space for the EMT training centre. Warrants that the site and premises of the Regional EMT Training Centre, as specified herein, may lawfully be used for such purposes and undertakes that WHO shall peaceably and quietly have, hold, and enjoy those premises for the duration of this MOU.	Contribute financially on a co-sharing basis for support services, including power and water. Provide security service for the Regional EMT-TC. Provide technical support to the Ethiopian field hospital and the national EMT in case of a request from the MoH. Ensure a backup system is in place for the installations dedicated to the Regional EMT-TC. Procure and maintain the office and training setup of the Regional EMT-TC. Assist the MoH in developing the National EMTs at national and sub-national levels with the possible engagement of members in the delivery of training to other teams within the premises of the EMT training centre. Respect the country’s cultural values, beliefs, norms, and traditions. Accept modifications to the common areas outside of the Regional EMT-TC premises.

Source: Draft MoU document, yet to be signed (under discussions) (27).

### Policy implementation

**Figure 3:**
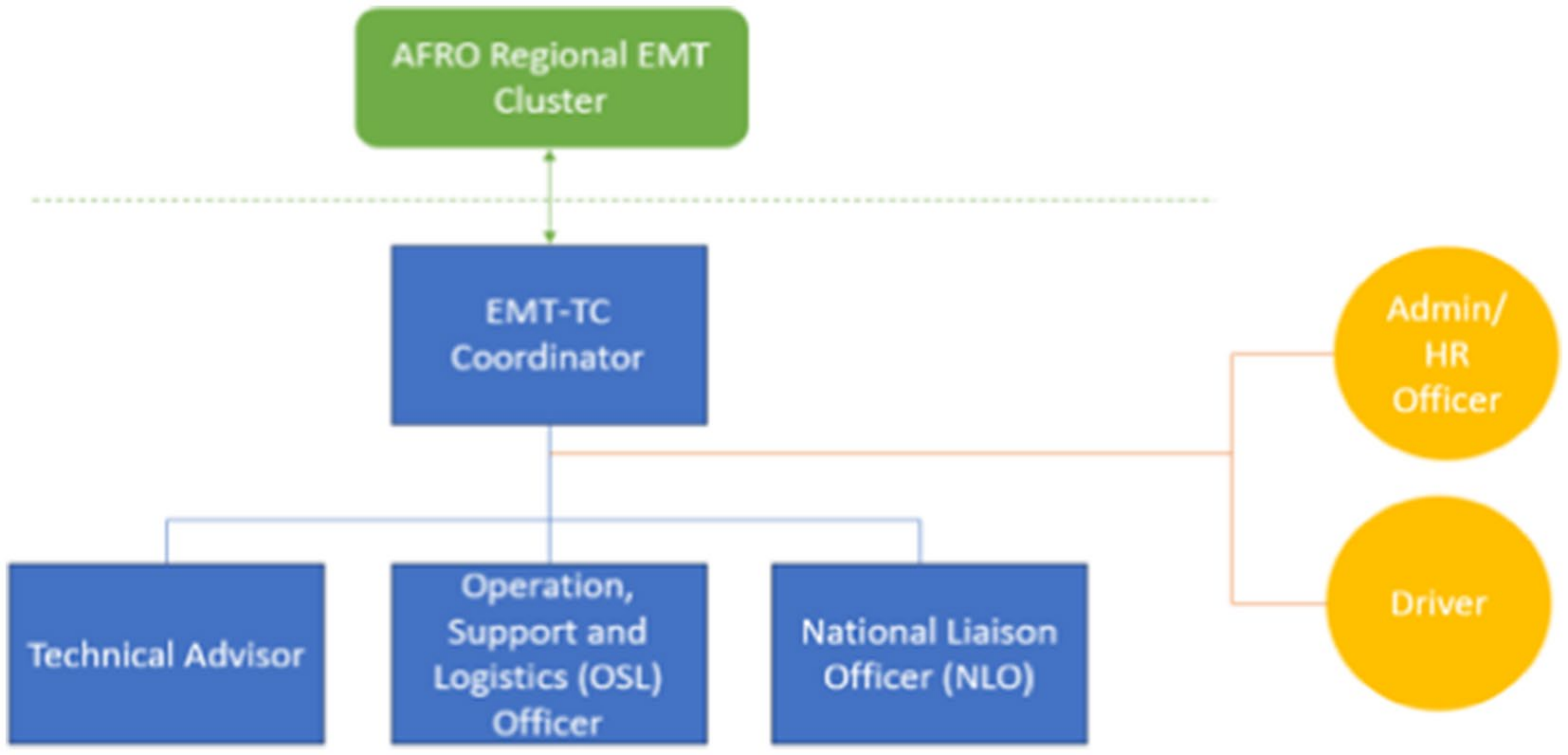
EMT-TC coordination as implemented.

***Management and coordination.*** The implementation started immediately following the joint official launch by the WHO and the MoH. During the implementation of the EMT training centre, the organogram was not executed as planned. Instead, a focal point was designated: a training coordinator who oversaw the entire training programme and managed the centre’s construction in different countries. The coordinator was based at the WCO in Ethiopia and was assisted by three core technical staff members: (i) Technical Advisor, (ii) OSL Officer, (iii) National Liaison Officer (NLO), as well as two support staff (see Figure 3).

***Training Planning and Management.*** Some of the trainings conducted by the regional faculty are presented in Table 4. Since implementing the training centre concept, 12 countries in 2022 and 7 in 2023 benefited from different training out of the 10 countries prioritised at the onset. The EMT TM induction used to provide a general overview of the EMT Initiative scope for National EMTs and to enlarge the roster of the country’s National EMTs has been conducted in 7 out of the 10 original prioritised countries (Togo, DRC, Mauritania, Namibia, Botswana, Uganda, and Ethiopia; Table 4). The envisioned training portfolio was executed as conceived and was not only limited to EMT focus but also captured a specific set of specialised EMT courses. Other specialised trainings, such as Case Management and Operation in Burns Mass Influx and EMT TM Induction, focus on cholera case management, which have also been conducted in countries that have faced such challenges. These were modifications of the originally targeted training. In total, there have been 460 participants who have benefited from the training (Table 4). Between October 2023 and September 2024, the Regional EMT-TC aims to provide 12 trainings to 360 health professionals. Additionally, seven awareness workshops were conducted between 2018 and 2020, and the EMT deployments to facilitate COVID-19 responses have been published elsewhere (6, 24).

**Table 4:** A selection of the trainings conducted by the regional faculty.

No	Country	Training	Date	Number of participants	Remarks
Trainings conducted in 2022					
1	Togo	EMT TM Induction	11/22	34	Training conducted in the country
2	RDC	EMT TM Induction	10/22	35	
3	Mauritania	EMT TM Induction	10/22	25	
4	Mauritania, Togo, Senegal, Guinee, Ivory Coast, Niger, Ethiopia, Namibia, Nigeria, and DRC	Case Management and Operation in Burns Mass Influx	10/22	33	Francophone and Anglophone sessions are done separately
Trainings conducted in 2023					
1	Namibia	EMT TM Induction	1/23	25	Training conducted in the country
		10-Step Implementation	1/23	20	
		SOP Workshop	10/23	15	
2	Botswana	EMT TM Induction	2/23	25	
		EMT TM Induction	11/23	25	
		10- Step Implementation	11/23	20	
3	Malawi	EMT TM Induction focus on Cholera CM	4/23	40	
		10- Step Implementation	6/23	30	
4	Uganda	EMT TM Induction	8/23	17	
		EMT TM Induction	8/23	25	
		EMT TM Induction focus on Cholera CM	9/23	25	
5	Cape Verde	EMT Awareness session	10/23	28	
6	Tchad	EMT TM Induction focuses on the Humanitarian Crisis	10/23	30	
7	Benin	Burn Management Specialised Training	12/23		
8	Ethiopia	mass casualty management			Training conducted by the WHO Academy at the EMT-TC
9	Ethiopia	EMT TM Induction	03/22, 06/22, 07/22	3	Training conducted at the EMT-TC
		10-step Implementation	03/22	1	
		Case Management Mentorship	09/23	1	
		Case Management Workshop	09/23	1	
		Burn Management Specialised Training	10/22	1	
		EMT ToT	02/22	1	

Rehabilitation training was planned for October in Ethiopia but has not yet been completed. Surgery in a humanitarian environment was also planned but has not yet happened.

While it was initially planned for all training to take place within the centre, most training sessions were held in hotels and countries such as Uganda and Namibia, although still under the supervision of the centre. Several factors contributed to this decision, including government requests for training and their ability to provide funding. In some cases, funding was also provided by WCO, as it was more cost-effective than sending participants to the training centre. Additionally, the speed at which issues were resolved between different WHO entities and other UN agencies involved in the construction process also influenced the location of the training.

**Figure 4:**
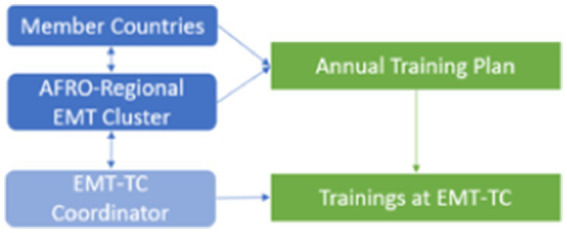
Procedure for planning and managing the training centre.

The EMT-TC Coordinator played a key role in organising and managing training operations in collaboration with the WHO AFRO Regional EMT Cluster. Together with member countries, the AFRO-Regional EMT Cluster developed and approved an Annual Training Plan, which served as the foundation for capacity-building activities. Based on this plan, the coordinator proposed specific training packages to interested member countries and ensured their successful implementation (see Figure 4). Additionally, efforts were made to establish a regional faculty team, which is now operational and comprises teams proficient in both English and French to enhance accessibility and effectiveness of training at the EMT-TC.

***Supporting the development of the national EMT.*** The EMT-TC has already made significant contributions since its establishment. The overall goal of the intervention was to train and form national and regional EMTs to promptly support national health systems during a surge period of health emergencies. As a result, countries like Ethiopia and Namibia are establishing their first national and/or regional EMTs, with six others in the advanced stages. This aligns with the 2030 plan that aims to implement at least one EMT in 70% of the countries in the region, with at least one Regional EMT in each of the regional languages spoken (English, French, and Portuguese) (28). The objective of developing fully trained national and regional EMTs capable of addressing health emergencies across the region would be achieved by supporting the intervention in making the EMT-TC fully functional and operational.

***Procurement and supply of equipment.*** There has been ongoing refinement throughout the implementation process. The procurement process began in August 2020 and is still ongoing. Initially, the list of equipment and consumables was redefined based on the needs and proposed scenarios from the scoping mission (22). The Ethiopian MoH provided initial equipment such as beds and ventilators. However, collaborative discussions between WHO HQ, AFRO, and the WCO led to the decision on the equipment and supplies needed to start the programme. The procurement discussions faced delays due to inter-agency discussions around training space, funding, and allocating procurement responsibilities.

Finalising the training and establishing a standardised equipment list proved challenging due to various logistical and political complexities. However, these inter-agency challenges were eventually resolved, allowing the process to move forward. In 2021, a comprehensive list of equipment and supplies was agreed upon, but further revisions and refinements were made during an EMT training discussion in January 2022. This collaborative effort involved the EMT teams at WHO HQ and AFRO, alongside the OSL teams from both WHO HQ and AFRO, ensuring that the final list was well-aligned with training and operational needs. This was subsequently published in annexe 2 of the technical note for WHO African Region Member States on building operational national EMTs in Africa (29). Regarding procurement, there were many things to consider when choosing training equipment and using the envisioned training portfolio (16), such as having different types of mannequins and airway equipment. Additionally, there was a need for a big field where tents would be set for the simulation training.

***Financing.*** The initial initiative’s financing was done by WHO and the EMT network partners, such as Norway, which played an important role, especially in the initial funding. Equally, the WHO AFRO Emergency Preparedness and Response cluster has provided funding to all the trainings for the different countries that have happened both at the different countries and the EMT-TC. The WHO HQ provided funding through the support of trainers who led technical subjects such as logistics and WASH. However, structured conceptions of each agency’s roles in the funding process are ongoing. The countries in the WHO African regions implementing their national EMTs are implementing strategies for resource mobilisation, including elements such as training and implementation of EMT activities. These have been done/ are being done through collaboration with partners, for example, in Namibia.

***Construction of the site.*** The actual construction of the centre was also faced with challenges. For instance, many issues with the land, power supply, and equipment made it unclear who was responsible for covering the daily subsistence of the different elements and who would manage the daily operations. Currently, the EMT-TC construction has been completed, and it is ready to be furnished and equipped. Once completed, the EMT-TC will reach its full capacity to provide training and simulation activities for EMTs across the region (25).

***Human resource.*** Initial staff involved in the concept have transitioned to different roles, posing a challenge. This has made it difficult to maintain momentum despite the support from various entities such as the Government of Ethiopia, the WCO, and AFRO. However, the regional faculty team has already been created and is fully operational in English and French (25). The faculty teams were selected from participants trained from different countries and who have undergone all the mandatory training, including the regional training coordinator and teams from the WHO AFRO and HQ who support backstopping for some of the technical teams based on their experiences from training and deployments.

### Policy evaluation

***At the onset of the training centre’s conception in October 2021, a comprehensive monitoring and evaluation (M&E) framework was developed to systematically track progress, assess implementation fidelity, and ensure continuous quality improvement.*** This framework was designed with a set of key performance indicators (KPIs) and structured evaluation components to measure training effectiveness, operational efficiency, and institutional impact. The development of these indicators was informed by global best practices in training programme evaluation, particularly drawing from WHO’s EMT performance assessment standards and other global health training frameworks.

During the scoping phase, teams from WHO HQ and the WHO AFRO collaboratively reviewed and refined the M&E framework, ensuring its alignment with regional training priorities and capacity-building objectives. The indicators were categorised into three core areas: operational readiness, which tracked the availability of training materials, faculty preparedness, and logistical planning; training delivery and participant engagement, which focused on course completion rates, participant competency improvement, and feedback mechanisms; and impact and institutional strengthening, which assessed long-term skill retention, the deployment of trained personnel, and the integration of learned competencies into emergency response protocols.

Despite the well-structured framework, implementation challenges arose due to severe staffing limitations at the training centre. At the initial phase, only one staff member was responsible for multiple operational tasks, including M&E functions. This resource constraint significantly weakened the ability to systematically collect and analyse data, making it difficult to measure the full impact of training activities. As one respondent noted, while the framework existed, the lack of dedicated personnel meant that M&E activities did not receive the attention they required.

*“…so…[only] one person who was tasked with so many other things that this monitoring and Evaluation [M&E] piece ended up not being as strong as we were hoping. But we did have a framework, we had structure, we had some indicators that we were tracking.” Respondent 01*

This staffing shortfall resulted in gaps in data collection, tracking, and performance evaluation, limiting the ability to fully assess training effectiveness and long-term impact. Recognising these limitations, steps have been taken to enhance M&E capacity.

### Future propositions of the training centre

The EMT centre is envisioned to expand beyond the initial scope and become a fully functional training centre. The following proposals are suggested:

a) Integrate the EMT Regional Centre as a hub for specialised training tailored to the needs of African Volunteers Health Corps—Strengthening, Utilisation, Readiness, and Governance for Emergencies (AVoHC-SURGE) experts—a rapid response team capable of deployment within 48 to 72 h. This centre would bridge training gaps that are currently unmet in various countries, providing a comprehensive platform for diverse capacity-building initiatives. It could facilitate multi-disciplinary training programmes, including Rapid Response Team training, AVoHC-SURGE preparedness sessions, and simulation-based exercises, ensuring that responders are well-equipped to handle emergencies effectively.

*“Use that regional EMT setup support on other aspects related to supporting the building of the EMT network, which the EMT coordinator could help to build. Considering that we are part of whatever is happening in AFRO regions, we want to ensure that the EMT Centre participates in some aspects.” Respondent 02*

b) It is envisioned as a knowledge hub, serving as a collaborative platform where the region and member countries can work together to enhance the effectiveness of their activities, strengthen interoperability, and foster communities of practice. Additionally, it will support the development of research initiatives and provide a space for stakeholders to share best practices and learn effective documentation methods.

*“To ensure that there is a culture of data collection and analysis regarding EMT work, which should be fitting within the EMT Regional Center [EMT-Training Centre]. Quality assurance and accreditations, especially for national EMTs, should be the responsibility of the regional EMT coordinator, and the other aspects are research and innovations.” Respondent 02*

c) Support existing and upcoming twinning programmes, such as the Namibia-German EMT partnership, the UK-MED collaboration with Ethiopian EMTs, and the Uganda-German EMT (Maltiser EMTs) twinning initiative.

*“I think the good thing about these twining programs is that whatever needs to be done, they should be using our EMT Center as a platform to support whatever the national EMT needs.” Respondent 02*

d) Ensure that EMTs are fully integrated into emergency preparedness efforts, with a focus on capacity-building and prepositioning essential kits and supplies before emergencies arise. For countries with established EMTs, the EMT-TC will provide customised training programmes tailored to their specific needs, enhancing their readiness for both current and future emergencies. Meanwhile, for countries that have not yet initiated the EMT development process, the EMT-TC will offer guidance and foundational support to facilitate their establishment and integration into national and regional emergency response frameworks.

*“Those are the just ways of maximising the EMT said that will request this holistic approach…and will be very supported to ensure that we can provide needed health care services, wherever needed in Africa regions…the EMT training coordinator should be able to provide us with the minimum equipment and materials needed for emergency response, and we have some specialised emergency kits. Most of the time, we may need these kits based on the different situations we are facing in Africa; we have cholera kits that support limited management for cholera cases, and we have a trauma kit which can be used to support any trauma situations where having been kits.” Respondent 02*

e) To strengthen the future operations of the training centre, it is essential to enhance and reorganise its management structure. While the WHO Country Office (WCO) and the Ethiopian government would oversee administrative functions, the overall management of the training centre should remain a collaborative effort with WHO. Additionally, exploring alternative management models, such as engaging a third-party organisation with expertise in the EMT approach, could be considered. This would be done in close collaboration with WHO, ensuring alignment with strategic regional objectives and enhancing the centre’s effectiveness in emergency medical training.
f) Strengthen the Centre’s Role in Building a Regional EMT Expert Pool—Leverage the centre to develop a skilled pool of regional EMT professionals drawn from existing national EMTs, ensuring readiness for deployment in support of EMT activities across the region. This pool should include specialists such as surgeons, anaesthesiologists, nurses, and psychologists, who can receive additional capacity-building training and be rapidly mobilised when emergencies arise.

*“We did it in Chad…We deployed people from five countries: DRC, Togo, Burkina Faso, and Senegal…So they came with different backgrounds, surgeons, anaesthesiologists, nurses, psychologists, it’s really regional EMT teams. So when deployed, they are doing great work. They’re in their second month now.” Respondent 03.*

g) Prioritise training and technical support to standardise emergency response capacities in alignment with newly developed guidance documents. This includes key thematic areas such as highly infectious diseases, medical evacuation, operations in insecure environments, maternal and child health, rehabilitation, and mental health and psychosocial support (MHPSS).

## Discussion

This paper used a policy cycle lens to describe the development and progress of the EMT training centre in the WHO African Region over the past 4 years. One key observation that emerged from exploring the agenda setting of the development of the training centre is that establishing an evacuation centre for UN staff at the beginning of the pandemic, along with subsequent political support, necessitated its creation. This analysis indicates that a ‘window of opportunity’ presented itself, where the policy (the need for a centre that evolved from an evacuation centre), the problem (gaps in strengthening the EMT’s knowledge), and political will (political support) converged at an opportune time, as noted by Kingdom (30). By focusing on agenda setting and the conception of such initiatives, the political significance of these reforms and the need to set the agenda to meet reform goals mirror the ongoing need to develop effective initiatives in the continent. Moreover, by shifting the perspective from the original need for an evacuation centre to the development of a training centre, this initiative is taking a flexible, fluid approach instead of remaining fixed in a static form. Similar findings have been observed in other global policy initiatives or theories (31–33).

The findings highlight that countries are actively strengthening their EMTs through targeted training, reflecting a strong commitment to capacity enhancement, particularly in light of the unique vulnerabilities within the African context. Additionally, the integration of EMT training into national emergency response strategies ensures long-term sustainability and reinforces resilience in managing public health emergencies. The number of training sessions has increased and so have the commitments from countries courtesy of the launch of the centre. This has been essential and incorporated into the regional strategy and aligned with the 2030 EMT strategy (24, 28), clearly highlighting the need to set up this type of facility to allow workforce growth and strengthen technical skills, having multi-country coming together and sharing experiences and testing each other, and having a centre for innovations and research contributing as well to the bigger vision of a global health emergency corps. Besides, the training benefits the health workforce because the trained workers work in clinics or hospitals daily. With the training on EMTs’ work, they can support the strengthening the resilience of health systems aligning with the overall WHO goal of strengthening the global architecture for health emergency preparedness, response and resilience (34).

The African continent experiences a diverse range of emergencies and disasters, including natural, weather-related, and human-induced events, with their impacts varying significantly by geography and location. The WHO EMT Training Centre plays a critical role in addressing these challenges by bridging capacity gaps and advancing pandemic preparedness, fostering a healthier, more resilient, and self-sufficient Africa. Through the centre, countries are prioritising specific needs and investing in the EMT approach to strengthen their ability to respond effectively to crises.

The centre aligns closely with the African Union’s Agenda 2063, WHO’s Health Emergencies Programme, the 2030 EMT Strategy, SDG 3 (Good Health and Well-being), and the WHO Regional Office for Africa’s priorities, focusing on building robust national, sub-regional, and regional capacities tailored to local contexts, including culture, language, and health systems. It supports the WHO’s flagship Regional Emergency Preparedness and Response programmes by enhancing operational readiness and ensuring sustainable capacity-building (35). By equipping EMTs with specialised training to manage pandemics, disease outbreaks, and natural disasters, the centre enhances technical expertise and promotes integrated surveillance and rapid response strategies, ensuring timely identification and containment of health threats. Aligned with Agenda 2063 (36), the centre contributes to Goal 3 (healthy citizens), Goal 17 (capable institutions), and Goal 12 (resilient systems) while fostering collaboration among African nations. Its initiatives also support Goal 7 (environmental resilience) and Goal 20 (self-reliance), reducing reliance on external interventions and empowering Africa to independently tackle public health emergencies. These efforts bolster resilient health systems, advance universal health coverage, and address equity gaps, particularly in underserved regions.

Our findings demonstrate that the training centre was established in Addis Ababa due to its strategic location and accessibility for training national EMTs. The aim was to enhance the technical and operational skills of health workers in the region and develop other clinical care management skills crucial in managing severely ill and critical patients using simulation centres. Knowledge about new clinical developments is often shared through webinars, online courses, or in a classroom setting. However, this approach is less effective in improving the technical skills of healthcare workers when used alone. Simulation-based training is superior to lecture-based training for acquiring overall situation awareness since it enhances perception ability (37). Simulations also improve success rates of first attempts of intubations in critically sick patients (38). Technical skills such as intubation, use of ventilators, interpretation of blood gas results, and patient monitoring are difficult (if not impossible) to teach without simulation. Providing technical skills to healthcare workers in the region can benefit from simulation-based training offered at designated centres (39).

The scoping mission was conducted to understand the situation before establishing the centre. The mission aimed to clarify the scope and role of all stakeholders involved in the project, as well as the role of the centre and the project coordinator. Following the mission, it was identified that the structure built by the WFP, covering an area of around 11,000 m^2^, required adjustments and support from the government to make the tent structure functional and operational for both the treatment and training centres. Effective capacity building requires an accurate understanding of the context, including existing capacities and needs. Sufficient time is necessary to conduct a proper analysis and engage in dialogue with local and regional actors.

While many member states have received face-to-face training on the EMT methodology, resources have been lacking to provide them with complementary training through simulation exercises. Various studies have shown that simulations lead to longer-lasting skills acquisition and retention when practised and sustained through refresher simulation courses. Establishing dedicated simulation centres in the two WHO Regions will ensure the consistent enhancement of technical skills using the methodologies and quality standards of care and expertise (40). Healthcare workers will have the opportunity to benefit from exposure to training and innovations that would not be otherwise available in their regular working environments. While it is important to train with the equipment available to team members in their respective teams (e.g., oxygen concentrators, ventilators), the training centre focuses on the training methodologies (e.g., simulations, manikin) and the testing of innovative solutions to support the health workforce’s commitment to serve affected populations.

The project leveraged the EMT network alongside WHO-collaborating centres, academia, and other WHO technical units to develop and implement comprehensive training programmes, curricula, evaluation criteria, and follow-up mechanisms. These partnerships played a crucial role in providing specialised human resources for targeted training initiatives, supporting train-the-trainer programmes across the region, and ensuring continuous evaluation of project implementation. Beyond its initial focus on the COVID-19 response, the initiative remains active, reinforcing the skills of the broader health emergency workforce and contributing to long-term health system resilience.

To sustain and expand this impact, continued investment in workforce development and capacity-building will be essential. Long-term financial commitments from governments, global health partners, and private sector stakeholders will be critical to ensuring the centre remains a catalyst for health system transformation and emergency preparedness. Strengthening collaborations with academic institutions, regional training hubs, and WHO-collaborating centres will further enrich curriculum development, facilitate knowledge exchange, and drive research innovation. Additionally, the integration of digital learning platforms and simulation-based training will enhance accessibility and effectiveness, equipping frontline healthcare workers with the latest skills and best practices. A multi-sectoral approach encompassing resource mobilisation, policy integration, and long-term sustainability planning will solidify the centre’s role in fortifying Africa’s resilience to public health emergencies and advancing global health security.

Our study is not without limitations. The lack of a fully implemented monitoring and evaluation framework may have restricted the ability to comprehensively measure the impact of the EMT training centre. Additionally, the limited number of key informant interviews may not fully reflect the diversity of perspectives and experiences related to the centre’s development and operations. Furthermore, the study’s reliance on limited secondary data may have constrained the depth of analysis and validation of findings. Despite these limitations, the study provides valuable insights into the centre’s role, challenges, and opportunities, offering a transparent and reflective assessment of its progress and impact. Future studies should incorporate a more robust monitoring system, expand the range of informants, and integrate primary data collection to enhance the comprehensiveness of findings.

## Conclusion

The WHO EMT Training Centre in Addis Ababa plays a transformative role in building Africa’s capacity to respond to public health emergencies. By shifting its focus from an evacuation facility to a specialised training hub, the centre has effectively capitalised on a policy window where need, opportunity, and political support converged. Through simulation-based training, it equips healthcare workers with critical technical and operational skills, such as intubation, ventilator use, and patient monitoring, which are difficult to acquire through traditional lecture-based methods.

These initiatives are in direct alignment with the African Union’s Agenda 2063 and WHO’s strategic objectives for health emergency preparedness, universal health coverage, and resilient health systems, with a particular focus on closing equity gaps in underserved and vulnerable regions. The centre is closely integrated with WHO’s Health Emergencies Programme, the 2030 EMT Strategy, SDG 3 (Good Health and Well-being), and the WHO Regional Office for Africa’s priorities. It is dedicated to strengthening national, sub-regional, and regional capacities while ensuring that training and response mechanisms are adapted to local cultural, linguistic, and healthcare system contexts. Additionally, the centre plays a crucial role in supporting WHO’s flagship Regional Emergency Preparedness and Response programmes by enhancing operational readiness and fostering sustainable capacity-building efforts across the continent.

The successful establishment and operationalization of the EMT Training Centre marks a significant milestone in Africa’s health security landscape. By serving as a regional hub for emergency preparedness, the centre has trained hundreds of healthcare workers, strengthened health system resilience, and contributed to a coordinated continental response capacity. Additionally, its strategic location and integration with WHO’s emergency response framework ensure that African nations benefit from a structured, locally driven approach to workforce development, reducing reliance on external emergency medical teams.

Despite its achievements, the centre has faced key challenges that need to be addressed for sustained success. Limited monitoring and evaluation mechanisms have hindered the systematic assessment of training outcomes, while infrastructure constraints, resource limitations, and staffing shortages have occasionally impacted operational efficiency. Strengthening these areas will be essential to maximising the long-term impact of the training initiatives and ensuring continuous quality improvement.

The need for sustained investment in training, infrastructure, and monitoring systems is paramount. Expanding disease simulation programmes, integrating digital learning platforms, and embedding research-driven evaluation processes will enhance the effectiveness of the training programmes. Strengthening partnerships with WHO-collaborating centres, academia, and regional health institutions will help develop a comprehensive, context-specific curriculum that reflects Africa’s diverse healthcare challenges. Further investment in workforce development and long-term funding commitments from governments and global health partners will ensure the centre remains a catalyst for health system transformation and emergency preparedness.

Going forward, scaling up disease simulations and embedding additional training programmes will be critical for sustained impact. Expanding partnerships with WHO-collaborating centres, academia, and local institutions will enrich curriculum development, strengthen evaluation mechanisms, and promote regional ownership. Further investment in context-specific training that considers cultural and resource variations will enhance the effectiveness of these initiatives. By prioritising capacity building and knowledge sharing, the centre positions Africa on a pathway towards a healthier, more resilient, and self-sufficient future in addressing public health challenges.

## Footnotes

^1^**Clinical:** Airway management, Oxygen therapy management, Screening & Triage, Resuscitation, Rehabilitation/ Step down, IPC Standard and Transmission-based precautions; **WASH:** Water supply, Environmental sanitation, Hygiene, Environmental cleaning, Waste management, Dead body management; **Logistics:** Safety & Security, Cold chain management, Oxygen supply, Power & Electricity, Warehouse management; **General:** Just In Time Training, Open WHO COVID-19; **Surveillance:** Data management and reporting; **Other:** Public Health related including Laboratories.
